# A three-dimensional habitat for *C*. *elegans* environmental enrichment

**DOI:** 10.1371/journal.pone.0245139

**Published:** 2021-01-11

**Authors:** Aurélie Guisnet, Malosree Maitra, Sreeparna Pradhan, Michael Hendricks

**Affiliations:** 1 Department of Biology, McGill University, Montreal, Quebec, Canada; 2 Integrated Program in Neuroscience, McGill University, Montreal, Quebec, Canada; University of Southern California, UNITED STATES

## Abstract

As we learn more about the importance of gene-environment interactions and the effects of environmental enrichment, it becomes evident that minimalistic laboratory conditions can affect gene expression patterns and behaviors of model organisms. In the laboratory, *Caenorhabditis elegans* is generally cultured on two-dimensional, homogeneous agar plates abundantly covered with axenic bacteria culture as a food source. However, in the wild, this nematode thrives in rotting fruits and plant stems feeding on bacteria and small eukaryotes. This contrast in habitat complexity suggests that studying *C*. *elegans* in enriched laboratory conditions can deepen our understanding of its fundamental traits and behaviors. Here, we developed a protocol to create three-dimensional habitable scaffolds for trans-generational culture of *C*. *elegans* in the laboratory. Using decellularization and sterilization of fruit tissue, we created an axenic environment that can be navigated throughout and where the microbial environment can be strictly controlled. *C*. *elegans* were maintained over generations on this habitat, and showed a clear behavioral bias for the enriched environment. As an initial assessment of behavioral variations, we found that dauer populations in scaffolds exhibit high-frequency, complex nictation behavior including group towering and jumping behavior.

## Introduction

Model organisms are reared in laboratory conditions that are simplified compared to their wild natural environments. These conditions aid in standardization, however as we learn more about the importance of gene-environment interactions and the effects of environmental enrichment, it becomes evident that minimalistic laboratory conditions can affect gene expression patterns and behavior in ways that limit rather than enable biological understanding [1, 2]. For instance, 80% of the baker’s yeast *Saccharomyces cerevisiae* genes can be deleted under optimal laboratory conditions, but 97% of its genes are essential to survive environmental stressors [3]. There is a tension between the desirability of simple, uniform conditions that support reproducibility and the artifactual nature of traits—particularly behavioral traits—that can arise in impoverished environments [4]. Growing evidence suggests that considering a broader range of environmental factors and their effects on traits of interest in model organisms can provide deeper biological insight [5]. Environmental enrichment, which involves adding features to animal housing that provide sensory stimulation or promote activity, exploration, and interaction, is a strategy to study how these factors contribute to biological traits of interest [6]. For example, mouse, zebrafish, and house cricket models have all demonstrated increased performance in memory tasks when reared in enriched conditions [2, 7, 8].

*Caenorhabditis elegans* has typically been studied under standardized laboratory conditions, and only recently has more attention been given to its ecology and natural history. In its natural habitat, this small, transparent nematode is found in rotting fruits or plant stems and feeds on bacteria and small eukaryotes [9]. Their ecological prevalence is affected by several biotic factors such as the presence of other nematode species, humidity, and rain [10]. The majority of individuals found in natural populations are in the dauer stage, an alternate, developmentally arrested stage of the animal’s life cycle [11]. The dauer stage is characterized in part by a thinner body and nictation—a behavior where nematodes stand straight on their tail and wave their body in all directions [12]. Nictation is thought to mediate dispersal between food sources by allowing nematodes to adhere to organisms such as slugs and millipedes when food resources are exhausted [13, 14]. While the genetic mechanisms regulating dauer have been studied in great detail, the dynamics of dauer entry, exit, and nictation in natural contexts, like many wild traits of *C*. *elegans*, remain to be completely understood. Environmental conditions in the wild are drastically different than in the laboratory. In standard laboratory settings, worms live on a two-dimensional, flat, homogeneous agar surface coated uniformly with abundant axenic bacterial cultures as a food source and are kept under stable humidity and temperature conditions. In addition, the dauer stage is not encountered in laboratory stocks maintained on food, but can be induced by starvation or high temperature [15]. While food is abundant, laboratory conditions are otherwise impoverished compared to the rich and heterogeneous three-dimensional natural environments where wild *C*. *elegans* live. Extending the range of controlled environments in which C. elegans can be cultured in the laboratory, and consequently their expressed behaviors, can allow us to better understand the mechanisms behind these behaviors [1].

The difficulty of studying nematodes in the wild and the restrictive standard laboratory setting mean that we are probably unaware of the true range of possible *C*. *elegans* behaviors and how these might be influencing underlying molecular and neuronal pathways studied [16]. Yet, most *C*. *elegans* research is conducted disregarding that the animal’s sensory and navigatory mechanisms are adapted to three-dimensional habitats. In an effort to bridge this gap, various methods have been tested to create more complex habitats for *C*. *elegans*. On two-dimensional agar plates, nematodes are subject to strong surface tension force, and lessening this force through the addition of a third navigable dimension has the potential to alleviate physical restrictions [17, 18]. For example, when swimming in liquid culture conditions, worms exhibit different patterns of neuromuscular activity than when crawling on a 2D surface [19]. Similarly, their speed can be increased up to ten-fold with specially designed microfluidic chambers [20]. However, liquid and gel substrates can have limitations when contrasting behaviors or genetic expression patterns under standard conditions. In particular, animals in liquid culture are constantly swimming while suspended in liquid and cannot exhibit foraging behavior [21]. In gel media, food is dispersed into patches and animals have to burrow tunnels to bridge those patches. In addition, these techniques and other 3D habitats, like using soil, molded agar, beads or gauze, do not allow for individual selection of animals over generations [12, 22, 23].

Here, inspired by Modulevsky and colleagues [24], we developed a protocol to create three-dimensional habitable scaffolds for multi-generational culture of *C*. *elegans* in the laboratory. By decellularizing and sterilizing fruit tissue, we created an axenic environment that can be navigated throughout and where the microbial environment can be strictly controlled. Because processed fruit tissue may bear some resemblance to the preferred natural habitats of fruit nematodes, we predicted that the environmental conditions of the three-dimensional scaffold would be perceived as favorable by *C*. *elegans* and engender behavioral changes. Indeed, *C*. *elegans* were maintained over generations on this habitat, and showed a clear behavioral bias for the three-dimensional scaffold. As an initial assessment of behavioral variations, we found that animals exhibited a broader range of locomotory patterns, and dauer populations in 3D scaffolds exhibit complex nictation behaviors, including group towering and jumping, at high frequency, suggesting this is an attractive platform for studying nictation.

## Results

### Protocol design

**Fig 1:**
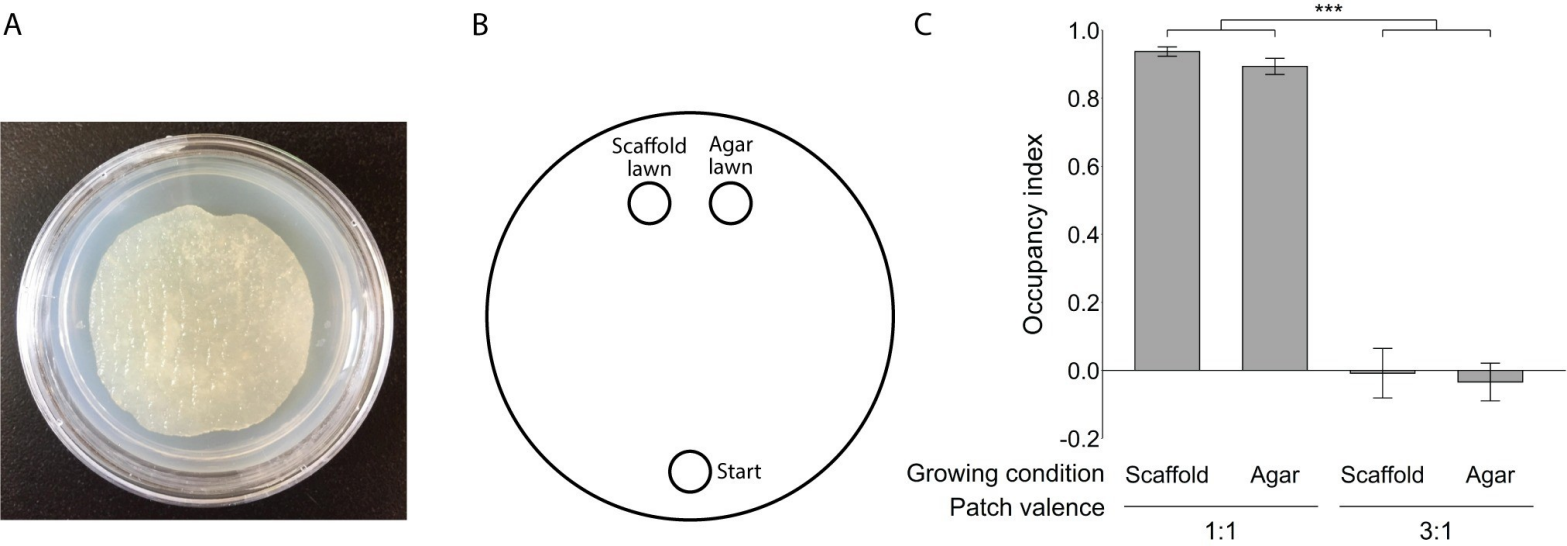
Habitat occupancy is independent of rearing condition. (A) Scaffold plate. (B) Occupancy experiment schematic. Animals were introduced at the start site on the opposite side of the food patches. The 2D and 3D patches were placed on the opposite side and 1 cm from each other. Animals were left to roam the plate overnight after which the number of animals in each location was counted. (C) Occupancy index for animals grown on scaffold plates and agar plates when (left) both patches had the same food concentration 1:1 patch valence and (right) when the 3D patch was diluted 3-folds compared to the 2D patch 3:1 patch valence (n = 20–30 animals per replicate, *** p < 0.0001, error bars are standard error mean).

The recent success of Modulevsky and colleagues [24] to use decellularized apple tissue to culture mammalian cells in three dimensions inspired our use of this tissue to culture *C*. *elegans* with an *E*. *coli* food source. We made apple tissue slices of approximately 3 mm thickness to allow animals to position their body fully vertically. We decellularized the tissue slices in sodium dodecyl sulfate (SDS), leaving behind only extracellular matrix comprising cellulose and other insoluble cell wall material. A series of water, ethanol and nematode growth medium (NGM) liquid washes were performed to wash out the SDS solution (see Materials and Methods). The bacterial culture was seeded uniformly through the scaffold by incubating scaffold slices in liquid bacterial culture. Standard agar plates were seeded with the same bacterial culture and incubated in parallel with scaffold slices. After incubation, scaffold slices were deposited on standard unseeded agar plates finalizing the three-dimensional habitat. Both plate types (“scaffold plates'' and “agar plates”) were sealed with parafilm to prevent drying (Fig 1A).

Our protocol resulted in a porous, navigable, structurally stable and habitable three-dimensional matrix. We were able to maintain over 30 generations of *C*. *elegans* in the new controlled three-dimensional habitat. We visualized bacterial growth in scaffold by seeding with an *E*. *coli* strain expressing GFP. This revealed that bacteria grew in an uneven, reticular pattern that appeared to follow the interstitial spaces between plant cell wall material. By adjusting incubation time and temperature, we obtained the similar density of bacteria per cross-sectional area between the two types of plates (see Materials and Methods).

*C*. *elegans* navigating through scaffold plates demonstrated crawling behavior, not burrowing or swimming. However, their locomotion pattern was not as characteristically sinusoidal, perhaps due to reduced surface tension and/or the heterogeneity of the scaffold (S1 Movie). Animals could not be observed if located more than one millimeter inside the scaffold because of the opacity of the extracellular matrix and the similarity of its color to the animal’s body. It was possible to approximately double the depth of sight by passing scaffolds through a vacuum chamber before introducing worms. Regardless, worms, worm tracks and eggs were rarely seen outside the scaffold (on the agar surface) before 3–4 days after the first L4 animals were introduced. Despite the initial time investment required for the production of the decellularized scaffolds, the simplicity of the preparation steps and storage of prepared scaffolds make the regular use of this protocol elementary. Animals can be either picked with a wire or a piece of the scaffold can be cut with a scalpel. Buffers can also be pipetted over the scaffold to collect animals and eggs.

### Scaffold occupancy is not affected by prior experience

To investigate potential habitat biases, we performed a simple occupancy experiment (Fig 1B). Animals started far from the target food patches to promote chemotaxis towards this general direction. We positioned one agar food patch and one scaffold food patch relatively close to each other in order for the two patches to be included in their local search area [25]. With this layout, animals can sense and explore both food patches before settling. We used a small number of individuals per replicate to limit any effects of resource depletion. Thus, we measured relative occupancy in the scaffold food patch, the conventional agar food patch, or neither (outside a patch).

We hypothesized that rearing condition would bias occupancy towards the familiar food experience [26, 27], but that qualities of the scaffold patch would lead a portion of agar-grown animals to settle in the 3D patch instead. Thus, we expected scaffold-grown animals to show a strong occupancy in the scaffold patch and little occupancy in the agar patch, and agar-grown animals to separate between the two patches. However, irrespective of their rearing condition, both scaffold-grown and agar-grown animals occupied the scaffold food patch significantly more (Fig 1C and S1 Fig). This demonstrates that animals are more attracted to or are retained in the enriched habitat by factors that are not affected by prior experience.

To examine further the perceived qualities of the three-dimensional habitat, we repeated the occupancy experiment with the scaffold bacterial patch diluted 3-fold compared to the agar patch (3:1 patch valence). We hypothesized that decreasing the quality of the scaffold patch by reducing its nutritional content would make the previously observed bias for the scaffold patch diminish [26], but that rearing condition would bias occupancy towards the familiar environment. Surprisingly, under these conditions, the same proportion of individuals settled in the scaffold patch and the agar patch (Fig 1C and S1 Fig). Again, these results were not affected by rearing conditions.

### 3D habitat promotes complex nictation behavior

*C*. *elegans* dauers cannot nictate on a flat agar surface, which creates difficulties for studying nictation [28]. They will however exhibit nictation if the agar is contaminated by fungus by climbing fungal hyphae [12]. They can also exhibit group nictation (towering or "tower of dauer") by climbing over each other if the agar plate is extremely crowded [29].

**Fig 2:**
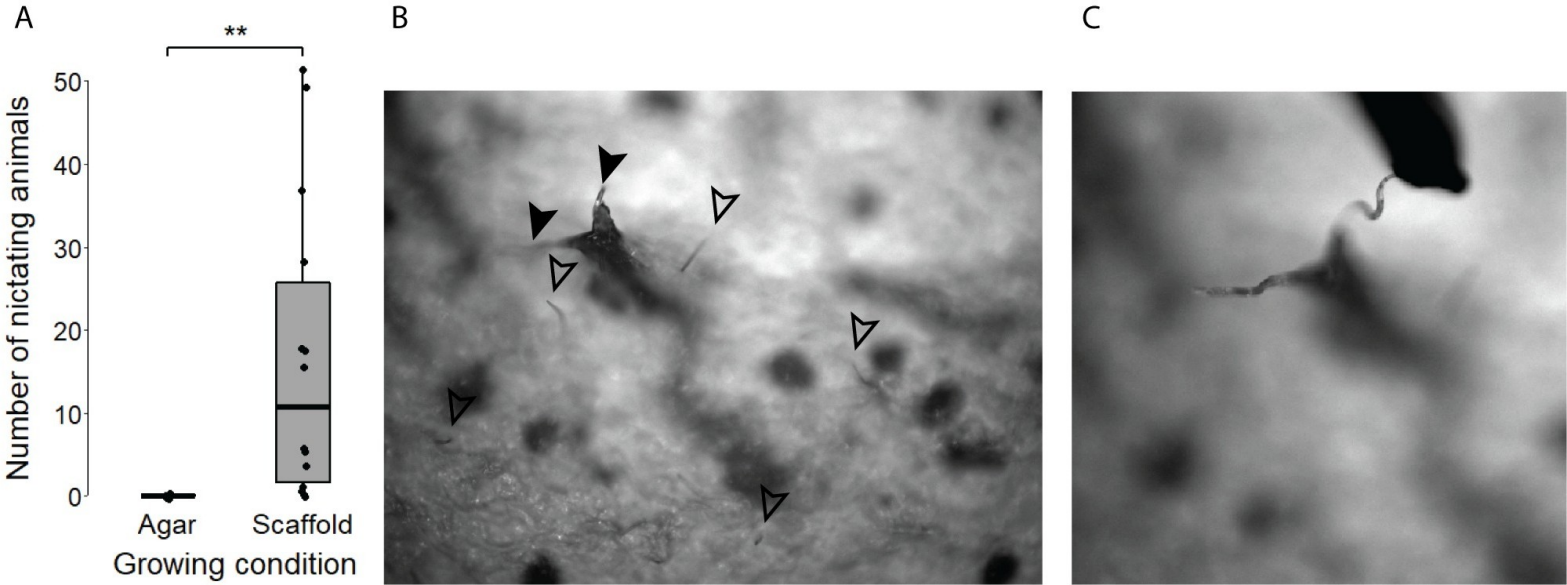
Nictation behavior is expressed only in 3D habitat. (A) Number of nictating 21-day old animals in the dauer stage by growing condition (agar-grown n = 12, scaffold-grown n = 14, ** = p<0.01, error bars are standard error mean). (B) Individual (hollow arrows) and group nictation (two animals, full arrows) on 3D habitat. (C) Nictating dauer jumping onto an experimenter’s pick.

We investigated the potential of our three-dimensional habitat for studying nictation by simply keeping inhabited plates at 20°C for several days. We expected dauers on the 3D habitat to be able to nictate due to the heterogeneity of the surface as previously described [12]. Indeed, we observed the most nictation events in 21-day old animals. In fact, although variable in number, we nearly always observed nictation on scaffold plates, but we never observed nictation on agar plates (Fig 2A). These results directly reinforce previous findings that the lack of nictation on agar is caused by physical limitations imposed by a smooth, two-dimensional substrate [12, 14, 22, 28].

Similarly to previous reports, we noticed that nictating individuals either waved their body in all directions or stood straight and immobile (Fig 2B, S2 and S3 Movies) [12]. In both situations, individually nictating animals had their tail anchored inside the scaffold. We were surprised to see jumping (“lashing”) behavior in nictating *C*. *elegans* at the approach of an experimenter’s pick, as this species of nematode has been previously reported to not have this behavior (Fig 2C and S4 Movie) [28]. Animals would sometimes adhere to both the pick and the scaffold at the same time. We also observed tower formation and collapse including anywhere from 2 to 30 individuals. Similarly to individually nictating animals, towers of dauers were either still or waving, and formed particularly on more acute portions of the scaffold, as previously reported in the wild [9]. Individuals forming towers were constantly moving and entangled together in a braid-like shape. These observations demonstrate that our habitat is permissive to the expression of behaviors that are repressed in standard laboratory conditions.

## Discussion

Conditions under which model organisms are reared in the laboratory can differ greatly from their natural habitat, and despite the advantages of simple and reproducible conditions, the effects of such impoverished environments can impede scientific discoveries [4, 30]. Here, we present a new effective and low-cost method to culture *C*. *elegans* in a structurally enriched three-dimensional habitat within controlled laboratory conditions. We showed that animals have a behavioral bias for and express complex nictation behavior as dauers in this habitat, including towering and jumping. We have yet to compare other phenotypic and behavioral traits, but no evident differences have been observed so far. Thus, we propose that the complexity of this living substrate has a significant effect on *C*. *elegans* behavior and that the basis of these behavioral changes and underlying mechanisms should be characterized further.

Several factors impact how *C*. *elegans* choose their food source including texture, nutrient content and abundance [31, 32]. They also have environmental preferences based on temperature and oxygen content [29, 33, 34]. We showed that, even when two food patches of equal nutritional content were provided, animals overwhelmingly occupied the 3D patch over the regular patch, revealing the importance of the perceived quality of the enriched habitat and suggesting a preference (Fig 1C). Yet, occupancy was equal between the 3D and 2D patch when food quality in the 3D patch was reduced three-fold (Fig 1C). This suggests that the perceived advantages of the 3D patch, such as decreased surface tension, increased navigable space and bacteria growth complexity, do not completely outweigh the importance of food availability. These results were not related to prior experience with the three-dimensional or two-dimensional habitat. Further investigation of the elements favored in the enriched environment has implications in characterizing *C*. *elegans* sensory abilities and distinguishing between a sensory attraction and an increased navigable space. Complemented with behavioral tracking, our method can contribute to understanding how *C*. *elegans* choose habitats and how early-life experience affects exploration patterns.

Nictation is an essential component of phoretic behavior for many wild nematodes [15]. We corroborate past observations that standard laboratory conditions are not permissive to the expression of this behavior (Fig 2A) [12, 28]. On the other hand, dauer populations on our three-dimensional scaffold showed consistent and complex nictation behaviors (Fig 2). An advantage of our habitat to study nictation behavior is that animals do not need to be moved to a new experimental system once they enter the dauer stage. As a result, dauer and nictation behavior can be studied in parallel at the population level and throughout development. We believe the expression of nictation behavior in the laboratory is enabled in part by the decreased surface tension and complex topography of heterogeneous surfaces like our porous habitat [17, 35]. In particular, the novel observation of jumping behavior suggests that this particular behavior in *C*. *elegans* must be particularly sensitive to environmental conditions. Nictation is a critical dispersal and survival behavior, and we believe this enriched habitat has the potential to enable its study in *C*. *elegans* and related nematodes.

Despite the difficulty of imaging animals inside the scaffold, we demonstrate that our habitat is usable for maintenance of *C*. *elegans* stocks, is preferred or promotes retention of animals compared to standard conditions, and offers a new tool for the study of nictation behavior in *C*. *elegans*. By developing a simple and convenient way to create enriched three-dimensional habitats, we provide an opportunity to expand research and consider findings in the natural living dimensions of *C*. *elegans* and other nematodes.

## Materials and methods

### Strains and culture

Wild-type N2 *C*. *elegans* were grown under standard conditions at 20°C and assayed at the early adult stage [36]. Worms were fed *E*. *coli* OP50-GFP. For all data reported, tested animals had been grown for more than 25 generations in their respective habitats (scaffold habitat or conventional agar habitat).

### Scaffold preparation

Freshly bought Macintosh apples (*Malus domestica*) were sliced with a mandolin at a thickness of 3.175 mm. Macintosh apples were chosen for their slow oxidation, moderate porosity for liquid exchange and moisture, average toughness, infrequent contamination, yearly accessibility and overall yield. Apples were kept for no more than 4 days at 4°C prior to use. Using a mold, slices were cut to a uniform 40 mm diameter round shape of hypanthium tissue while discarding the skin and slices containing apple ovary core. Freshly cut slices were decellularized by soaking overnight (12–24h) on a shaking platform in 0.5% SDS, followed by a series of washes at a ratio of 10 slices per liter of liquid were performed to remove the SDS and dehydrate the resulting cellulose scaffold in ethanol. The following steps were performed under sterile conditions: three brief washes in deionized water, two 30-minute washes in deionized water on an orbital shaker, one wash in 70% ethanol for one hour, and finally one wash in 100% ethanol for one hour.

Immediately after the last wash, each slice was individually vacuum sealed in sterile sample bags without removing the ethanol and kept at 4°C for storage. These were kept for no more than 3 weeks prior to use, and no degradation was visually observable in that time period.

### Plate seeding

We optimized the following steps to obtain equal cross-sectional density of bacteria on both regular agar plates and cellulose scaffold plates. To estimate cross-sectional density of grown bacteria, we extracted a vertical sample from each type of plate using a glass pipette. We streaked the resulting serial dilutions on LB agar plates and incubated them overnight at 37°C after which we compared the cfu by area. We compared several conditions of bacterial growth by changing incubation time, temperature and LB/NGM bacterial culture media. Scaffold plates systematically showed a lower cross-sectional density of bacteria, and frequent drying during incubation periods. Thus, we used NGM cultures (in which bacteria grow less densely) to reduce cross-sectional density in regular agar plates and submerged the cellulose scaffolds in the bacterial culture to promote growth throughout while preventing drying.

To remove ethanol, each slice was soaked in NGM liquid overnight on a shaking platform at a ratio of 1 slice per 50 mL of liquid. Scaffold slices were individually placed in a 6cm empty sterile petri dish submerged in NGM OP50-GFP culture (about 5 ml). Simultaneously, regular NGM agar plates were seeded with 100μl of NGM OP50-GFP culture. Both submerged slices and seeded agar plates were placed in a tightly closed box that was kept humidified with wet Kimwipes. The box was incubated at 28°C for 5 hours.

After incubation, each scaffold slice was placed on an unseeded NGM agar plate and sealed with laboratory film. Similarly, the incubated seeded agar plates were also sealed with laboratory film. Both were kept for at least 24 hours upside-down in a tightly closed box at laboratory room temperature (20°C) before use. All plates were used within three weeks (Fig 1A).

We established that it was necessary to seal cellulose scaffold plates prior to use to maintain moisture and prevent quality loss. Since bacteria metabolism is affected by gas exchange, we also sealed regular plates to have uniform conditions [32]. Also, we observed that keeping plates upside-down slowed drying and helped maintain scaffold integrity probably due to cellulose scaffold porosity and agar absorbability.

### Bleaching

To bleach scaffold-grown worms, scaffolds were first cut in three parallel pieces. The pieces were superimposed and moved to a side of the petri dish. 2 mL of bleach solution (2 bleach: 1 5M NaOH) was spurted over the cut scaffold while holding the plate at an opposite angle such that animals and eggs would be washed out and collected on the opposite side. This step was repeated several times (while avoiding reintroducing eggs and worms) to ensure that as many worms and eggs as possible exited the scaffold. Next, the agar was scraped with the pipette tip to detach fixed worms and eggs. Finally, eggs in bleach solution were rinsed three times with Milli-Q water following standard bleaching protocol [37].

### Occupancy experiment

In order to assess biases for habitat substrate, we designed a simple occupancy experiment (Fig 1B). We seeded two patches of 10μl lyophilized LabTIE International OP50 culture on one side of a large NGM agar plate: one on a 1 cm diameter piece of cellulose scaffold and one directly on the agar spread to be the same size as the scaffold (1:1 patch valence). The 2D and 3D patches were 1 cm apart. One hour after seeding, 20 to 30 young adult worms were placed at the start position and left to explore the plate overnight at room temperature. We then counted animals outside of either food patch and in the regular patch, and subtracted all of these from the initial number of animals to deduce how many were in the scaffold. We repeated this experiment for animals grown on regular agar plates and those grown in a cellulose scaffold for several generations. To create a lower quality food patch on the scaffold, we diluted the dried OP50 culture three-fold in water (3:1 patch valence). The occupancy index was calculated for every replicate of the task.

$$Occupancy\;index=\frac{\left(\#in\;3D\;patch-\#in\;2D\;patch\right)}{\left(Total\#scored-\#outside\;either\;patch\right)}$$

Occupancy index score ranges from -1 to +1. A more positive index indicates higher occupancy of the 3D patch whereas a more negative index indicates a higher occupancy of the 2D patch.

### Nictation

Both agar and scaffold plates were kept at 20°C after being used for worm maintenance. After 21 days, the number of nictating dauers were counted by eye. Because of an overwhelming number of nictating individuals on scaffold plates, nictation was assessed in an area made of a 1cm large strip drawn across the diameter of each plate. Each plate was counted independently by two experimenters and the average was used. Nematodes forming towers of dauers were also counted as nictating individuals. Agar and scaffold plates that were cracked or contaminated were not included in the count.

### Statistical analyses

All statistical analyses and graphs were generated with the R statistical software [38]. *t-test* with equal variance was performed between agar-grown group and scaffold-grown group for the nictation count. Three-way ANOVA test was performed for the occupancy experiment followed by post-hoc Tukey-HSD. Significance was assessed at *p* < 0.05.

## Supporting information

S1 Fig Bias for 3D habitat is lost when food quality is diminished. Percent of individuals by location after occupancy experiment for scaffold-grown and agar-grown *C*. *elegans* when (left) both patches had the same food concentration 1:1 patch valence and (right) when the 3D patch was diluted 3-folds compared to the 2D patch 3:1 patch valence. Color legend for bars and points denotes location (n = 20–30 animals per replicate).
(PNG)

S2 Fig (PNG)

S1 Movie Crawling *C*. *elegans* in 3D habitat. (MP4)

S2 Movie Waving and immobile nictating *C*. *elegans* dauers. (MP4)

S3 Movie Waving *C*. *elegans* dauer ending nictation. (AVI)

S4 Movie *C*. *elegans* jumping onto an experimenter’s pick. (MP4)

S1 Dataset Occupancy experiment dataset. (XLSX)
