# Supplementary figures and images for: A three-dimensional habitat for C. elegans environmental enrichment
Source: PLoS One. 2021 Jan 11;16(1):e0245139. doi: 10.1371/journal.pone.0245139 (PMC7799825; doi:10.1371/journal.pone.0245139)

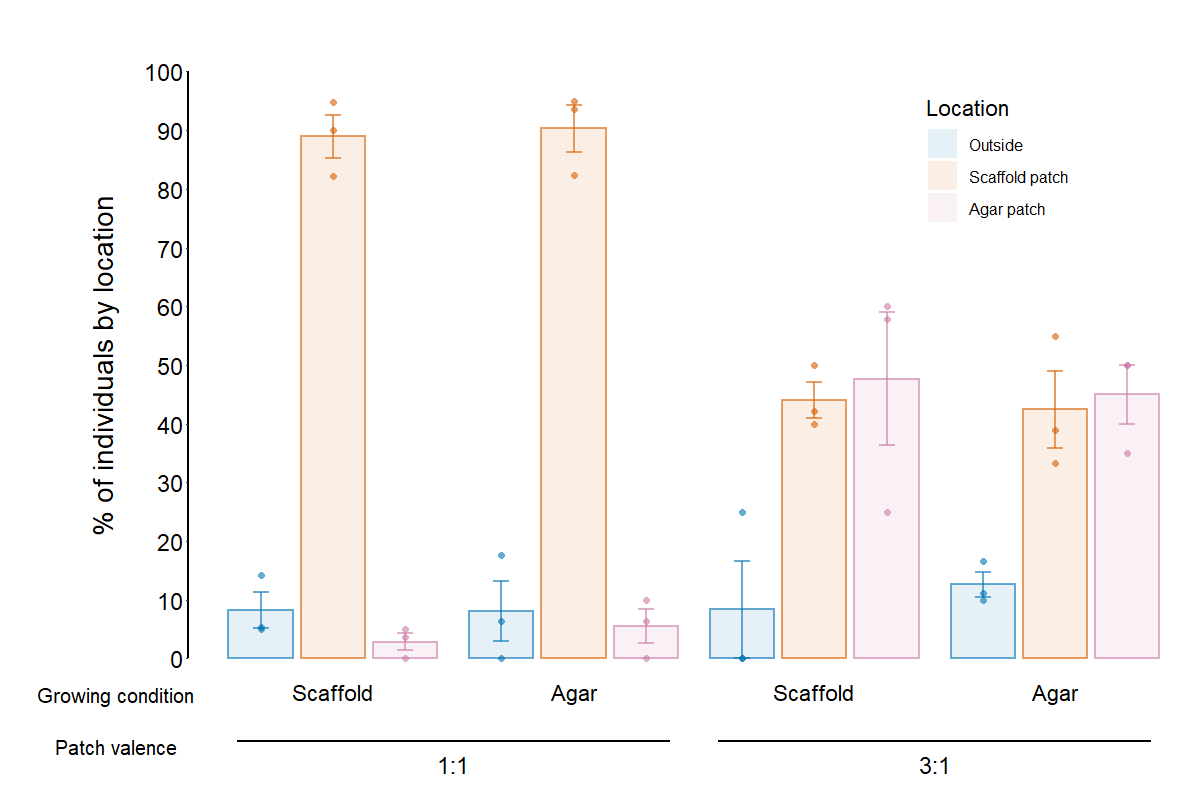

Supplement: S1 Fig — Percent of individuals by location after occupancy experiment for scaffold-grown and agar-grown C. elegans when (left) both patches had the same food concentration 1:1 patch valence and (right) when the 3D patch was diluted 3-folds compared to the 2D patch 3:1 patch valence. Color legend for bars and points denotes location (n = 20–30 animals per replicate). (PNG) [file pone.0245139.s001.png]

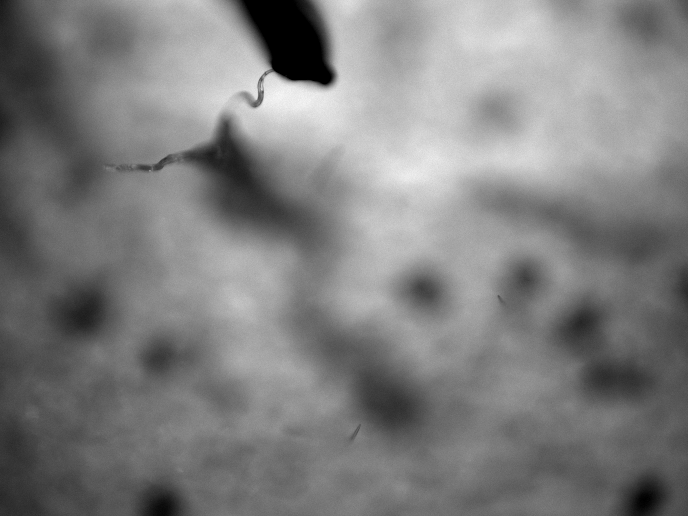

Supplement: S2 Fig — (PNG) [file pone.0245139.s002.png]
